# Are antimicrobial defences in bird eggs related to climatic conditions associated with risk of trans-shell microbial infection?

**DOI:** 10.1186/1742-9994-11-49

**Published:** 2014-07-02

**Authors:** Nicholas PC Horrocks, Kathryn Hine, Arne Hegemann, Henry K Ndithia, Mohammed Shobrak, Stéphane Ostrowski, Joseph B Williams, Kevin D Matson, B Irene Tieleman

**Affiliations:** 1 Animal Ecology Group, Centre for Ecological & Evolutionary Studies, University of Groningen, P.O. Box 11103, 9700 CC Groningen, The Netherlands; 2 Department of Zoology, University of Cambridge, Downing Street, CB2 3EJ Cambridge, UK; 3 Department of Ornithology, National Museums of Kenya, PO Box 40658, Nairobi, Kenya; 4 Biology Department, Science College, Taif University, P.O. Box 888, 21974 Taif, Saudi Arabia; 5 Wildlife Conservation Society, 2300 Southern Boulevard, 10460 Bronx, NY, USA; 6 Department of Evolution, Ecology & Organismal Biology, Ohio State University, 43210 Columbus, OH, USA

**Keywords:** Antimicrobial, Aridity, Humidity, Egg albumen, Lark, Lysozyme, Ovotransferrin

## Abstract

**Introduction:**

All bird eggs are exposed to microbes in the environment, which if transmitted to the developing embryo, could cause hatching failure. However, the risk of trans-shell infection varies with environmental conditions and is higher for eggs laid in wetter environments. This might relate to generally higher microbial abundances and diversity in more humid environments, including on the surface of eggshells, as well as the need for moisture to facilitate microbial penetration of the eggshell. To protect against microbial infection, the albumen of avian eggs contains antimicrobial proteins, including lysozyme and ovotransferrin. We tested whether lysozyme and ovotransferrin activities varied in eggs of larks (*Alaudidae*) living along an arid-mesic gradient of environmental aridity, which we used as a proxy for risk of trans-shell infection.

**Results:**

Contrary to expectations, lysozyme activity was highest in eggs from hotter, more arid locations, where we predicted the risk of trans-shell infection would be lower. Ovotransferrin concentrations did not vary with climatic factors. Temperature was a much better predictor of antimicrobial protein activity than precipitation, a result inconsistent with studies stressing the importance of moisture for trans-shell infection.

**Conclusions:**

Our study raises interesting questions about the links between temperature and lysozyme activity in eggs, but we find no support for the hypothesis that antimicrobial protein deposition is higher in eggs laid in wetter environments.

## Introduction

Microbial infection of eggs represents one of the main threats to the avian embryo during development [1], but the severity of this threat varies with environmental conditions. Microbial loads on eggshells and trans-shell infection rates are highest in cool, wet and humid environments. Consequently, declines in egg viability due to infection are greater and occur more rapidly in mesic and tropical environments than in drier, hotter, and more arid locations [2-6]. This might be because, relative to arid habitats, wetter environments are associated with increased primary productivity [7], including greater microbial abundance and diversity. For example, the abundance of microbes in soil, although strongly influenced by pH [8,9], is positively associated with precipitation [10-14], and soil microbes contaminate both birds and their eggs [15-17]. Bacterial loads in nests also correlate positively with precipitation [18], and birds in a temperate environment carry more microbes on their body and feathers than do related species in the desert [19]. Increased humidity can also encourage trans-shell infection, because in addition to promoting microbial growth on the eggshell [20-22], water is required to transport microbes through shell pores [23,24]. Conversely, the reduced moisture, increased solar radiation and temperature extremes associated with arid environments likely act to limit microbial assemblages [19,25-28] and microbial growth on eggshells [20-24]. Since the abundance of microbes on the eggshell is positively correlated with the probability of trans-shell infection [3,5], eggs laid in these settings may be at reduced risk of becoming infected.

To minimise infection by microbes, eggs possess physical barriers such as the shell and membranes, and chemical barriers in the form of antimicrobial proteins and peptides [29,30]. These barriers are set by the mother during egg formation, allowing her to transmit her experience of the wider environment to her offspring, thereby influencing offspring phenotype and survival [31,32]. Concentrations of antimicrobial proteins in the albumen relate to plasma levels in the mother [33] and are also related to her infection status [34,35]. Thus, mothers could adjust the level of antimicrobial defences they deposit in their eggs in order to optimise protection from trans-shell microbial infection [36-39]. Past studies of the antibiotic properties of eggs are few, and found no relationship between antimicrobial deposition and risk of infection within clutches ( [36,38], but see [33]). This could indicate that the costs of producing antimicrobial proteins are insufficient to require differential deposition, at least within clutches. However, concentrations of antimicrobial proteins vary considerably among species [36] and variation in the risk of trans-shell infection may be greater for eggs laid in different environments than for eggs in the same clutch within an environment [3]. A recent study of chickens showed that individuals exposed to greater pathogenic load did modify some aspects of egg defences compared to those housed under more sterile conditions [39]. We hypothesised that if antimicrobial defences have evolved to match the risk of microbial infection [36-40] then concentrations of antimicrobial proteins in eggs should vary with those environmental conditions that predict the risk of trans-shell infection.

To test this hypothesis we collected eggs from larks (*Alaudidae*) along a gradient of environmental aridity that ranges from hyper-arid to mesic [41-43]. Larks are an ideal system for our purpose because different species inhabit environments with different macroclimates and show a range of well documented physiological and life history traits associated with these environmental differences [19,41-43]. Thus, our arid-mesic gradient encompasses larks from hot and cold deserts, temperate pastures and tropical grasslands. All lark species build open-cup nests in open habitats, which might make them more vulnerable to microbial contamination than eggs of cavity-nesting species ( [44] but see [45]). Furthermore, all species commence incubation upon laying of the penultimate or last egg (see Additional file 1 for information on clutch sizes and onset of incubation), suggesting that the effects of early incubation in reducing risk of trans-shell infection are minimal in our study species [4,5,21].

We measured concentrations of lysozyme and ovotransferrin, the two most abundant antimicrobial proteins present in the albumen [46]. Lysozyme catalyses the lysis of cell walls of gram-positive bacteria [47]. Ovotransferrin has bactericidal properties and binds iron to make it unavailable for bacterial growth [48]. The antimicrobial activity of both proteins might be influenced by the pH of the albumen, with more alkali albumen being more bactericidal [49]. Therefore we also recorded the pH of the albumen. We predicted that concentrations of lysozyme and ovotransferrin would be lowest in eggs from more arid environments (those that are hotter and/or drier) and higher in eggs from more mesic locations (those that are cooler, wetter, or more humid).

## Results

**Figure 1:**
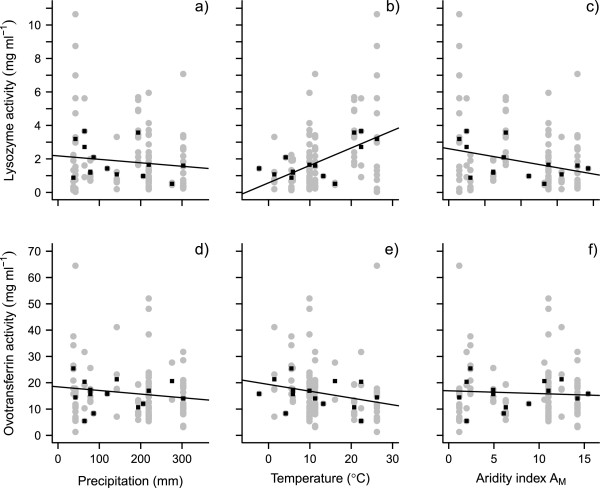
**Variation in antimicrobial protein activities in eggs from nine lark species in relation to precipitation, temperature and aridity, during the breeding season.** Activities of lysozyme and ovotransferrin respectively in relation to **(a and d)** mean precipitation, **(b and e)** mean ambient temperature and **(c and f)** the aridity index A_M_ during the breeding season. A lower value of A_M_ indicates a more arid environment. Raw data values are shown in grey, and mean values (based on mean values per nest) for each population are shown in black. The line of best fit is generated from a general linear mixed model containing only the variable of interest as predictor.

Along our arid-mesic gradient, eggs laid in wetter environments did not differ in their lysozyme activity from those laid in drier locations (Figure 1a and Table 1). However, eggs laid in hotter environments had higher lysozyme activity than those laid at cooler sites (Figure 1b and Table 1). Model averaging showed mean ambient temperature during the breeding season to be the best predictor of lysozyme activity (Table 2). Precipitation, albumen pH and our aridity index A_M_ all explained little of the variation in lysozyme activity among species (Figure 1a and c; Table 2). Precipitation, temperature and aridity were all poor predictors of ovotransferrin activity (Figure 1d-f; Table 1) and most of the variation in ovotransferrin activity could be explained by albumen pH alone (Table 2). Lysozyme and ovotransferrin activities appeared negatively correlated with each other, but this relationship was not significant, either at the level of individual eggs (Spearman’s rank correlation rho = -0.15 p = 0.09), or at the level of populations (rho = -0.37 p = 0.20).

**Table 1:** Generalized linear models investigating the influence of climatic variables and pH on antimicrobial protein activities

**Response variable**	**Model formula**	***k***	**∆AICc**	***w***_***i***_
Lysozyme	Temperature	5	0.00	0.484
	Temperature + pH	6	1.98	0.180
	Precipitation + Temperature	6	2.05	0.173
	Precipitation + Temperature + Precipitation:Temperature	7	3.78	0.073
	Precipitation + Temperature + pH	7	4.18	0.060
	Precipitation + Temperature + pH + Precipitation:Temperature	8	5.96	0.025
	A_M_	5	10.90	0.002
	Null model	4	11.39	0.002
	A_M_ + pH	6	12.95	0.001
	Precipitation	5	13.14	0.001
	pH	5	13.57	0.001
	Precipitation + pH	6	15.31	0.000
Ovotransferrin	pH	5	0.00	0.334
	Temperature + pH	6	0.29	0.289
	A_M_ + pH	6	2.09	0.118
	Precipitation + Temperature + pH	7	2.19	0.112
	Precipitation + pH	6	2.22	0.110
	Precipitation + Temperature + pH + Precipitation:Temperature	8	4.42	0.037
	Precipitation + Temperature	6	14.84	0.000
	Temperature	5	16.14	0.000
	Precipitation + Temperature + Precipitation:Temperature	7	17.07	0.000
	Null model	4	17.44	0.000
	Precipitation	5	17.99	0.000
	A_M_	5	19.40	0.000

Models are ranked by ∆AICc, which indicates the difference between each model and the model with the lowest AICc. The AICc weight, *w*_*i*_, illustrates the relative likelihood of a model given the set of candidate models, normalised to sum to one. *k* refers to the number of parameters in each model.

**Table 2:** Results of model averaging

			**95% CI**		
**Response variable**	**Model average parameters**	**Estimate (se)**	**Lower**	**Upper**	**Relative variable importance**
Lysozyme	Temperature	1.57 (0.39)	0.80	2.34	0.99
	Precipitation	-0.13 (0.37)	-0.85	0.58	0.33
	pH	-0.15 (0.35)	-0.83	0.53	0.27
	Precipitation:Temperature	0.80 (1.10)	-1.36	2.96	0.10
	A_M_	-0.92 (0.51)	-1.92	0.08	0.00
Ovotransferrin	pH	-8.14 (1.79)	-11.66	-4.62	1.00
	Temperature	-2.69 (2.00)	-6.61	1.23	0.44
	Precipitation	-0.78 (2.00)	-4.70	3.15	0.26
	A_M_	0.69 (1.84)	-2.90	4.29	0.12
	Precipitation:Temperature	-1.68 (5.62)	-12.70	9.34	0.04

Model average parameter estimates, standard errors (se), 95% confidence intervals and relative variable importance, demonstrating the effects of climatic variables and pH on antimicrobial protein activities. Relative variable importance is the sum of all AICc weights (*w*_*i*_ in Table 1) over all models containing the explanatory variable of interest. Effect sizes have been standardized on two SD following Gelman 2008 [50].

## Discussion

Immunological variation observed among individuals, populations and species might be a function of the risk of infection or disease, and variation in exposure of eggs to microbes has been suggested as one reason why birds might differentially deposit antimicrobial proteins into their eggs [36-40]. In related lark species, we found little evidence that climatic conditions associated with the risk of trans-shell infection could explain levels of antimicrobial proteins in eggs. We predicted that antimicrobial protein levels would be greater in eggs laid in wetter environments due to higher microbial abundances associated with increasing humidity [10-14,18,19] and the importance of water for facilitating passage of microbes through the shell [23,24]. However, we found no relationship between antimicrobial activities in lark eggs and any of the climatic variables that related to moisture. This result appears at odds with the work of Cook *et al.*[3-5] who reported higher trans-shell infection rates in wetter environments. Although moisture on the eggshell is important for microbial growth [20,21], more recent studies have found that bacterial loads on the eggs of a temperate, cavity-breeding passerine show no relationship with precipitation [51], but do correlate positively with relative humidity in the nest [22]. This suggests that precipitation does not predict moisture on eggshells and is not directly linked to risk of infection (but see [6,44,45]). Given that brooding birds shelter their eggs from rain, and incubation counters the positive effects of water on trans-shell infection [4,20-24], this is not surprising. Future work should focus on the nest microenvironment, as mechanisms of trans-shell infection at the level of the egg likely act differently to those functioning at the macro-climatic scale investigated in this study.

We found a positive correlation between lysozyme activity and temperature, but there was no effect of our aridity index A_M_, or the interaction of precipitation and temperature, on antimicrobial protein levels. Thus, contrary to our prediction, eggs laid in hotter, more arid locations did not have reduced levels of antimicrobial proteins. It is difficult to consider the effects of temperature in the absence of moisture, but higher bacterial growth rates in warmer conditions might predict a positive association between temperature and antimicrobial defences. However, at high temperatures, such as those experienced at the extreme end of our aridity gradient, bacterial growth may be inhibited [52]. Current evidence for a relationship between egg lysozyme and temperature is equivocal [53,54], and the small sample sizes in this study mean we must be cautious in interpreting the data. Nonetheless, this positive association between temperature and lysozyme activity is intriguing, not least because it is difficult to explain. Egg temperature is strongly influenced by incubation behaviour, which in turn can modify the risks of microbial infection [4,5,20-22], so links to ambient temperature might be expected to be weak. One possibility is that high lysozyme levels may be more important for eggs laid in hotter environments due to the physicochemical properties of lysozyme in eggs. Binding of lysozyme to the protein ovomucin determines albumen viscosity [55,56], which correlates positively with hatchability [57], but declines faster at higher temperatures [58]. Hence, high lysozyme levels in eggs laid in hotter environments might be unrelated to the antimicrobial functions of this protein.

Lysozyme and ovotransferrin showed weakly opposing patterns in relation to temperature, which could indicate a trade-off between these proteins, despite their negative correlation being non-significant. Lysozyme degrades gram-positive bacteria, while the iron-binding function of ovotransferrin makes it effective against a wide range of microbes. Since gram-positive bacteria tend to colonise egg contents less frequently [3-5], they may pose less of a threat to egg viability than other microbes, which could suggest that ovotransferrin is more valuable as a defensive protein than lysozyme. However, lysozyme can potentiate the activity of ovotransferrin and the two proteins may work in synergy, increasing their range of antimicrobial activity [59]. Thus, a balance between concentrations of lysozyme and ovotransferrin might be most effective in providing efficient, non-specific antimicrobial defence. This might also be preferable to simply increasing the amounts of either of these proteins in the egg, despite the fact that production costs may be minimal [33,36]. Increasing egg temperature by incubation provides an effective way of optimising antimicrobial activity [60], and other proteins and peptides also possess antimicrobial properties [30]. As more minor constituents of the egg, it may be easier to modify amounts of these other proteins in order to match egg antimicrobial defences to the risk of infection. In contrast, increasing amounts of lysozyme and ovotransferrin could be more difficult, since this might require a reduction in other aspects of albumen quality such as nutrition for the developing embryo. Experimental studies in chickens showing that egg white antimicrobial properties can be altered without changing lysozyme and ovotransferrin activities lend support to this idea [39,61,62]. Measuring the antimicrobial activity of whole albumen directly, in addition to concentrations of individual antimicrobial proteins, will yield additional insights. However, this will require further research to define suitable microbial challenges. Central to this work is the need to better describe and quantify the microbial assemblages of eggs and their microenvironments. Bacteria commensal with eggshells may be protective or pathogenic [4,17,63] but there is currently little information from natural systems on which strains reduce hatching viability [64], and whether these strains are ubiquitous among species and nest environments. Abiotic factors such as pH and salinity, that are important in determining microbial biogeography [8,9,65], should also be investigated for their role in shaping bacterial and fungal communities in nests and on eggs. Such knowledge is essential to developing a better understanding of the risks of trans-shell infection in different environments and will be informative in explaining how birds modify the infection risks to their eggs.

## Methods

### Antimicrobial protein assays

We collected 124 eggs from nine lark species in 12 climatically distinct locations (Table 3). Four populations had low sample sizes (fewer than two eggs or nests per location; Table 3) but excluding these populations did not qualitatively alter our findings and so we present all results with these populations included. Eggs were collected on ice and dissected into constituent parts on the day of collection (n = 32) or where this was not possible, were stored whole at -20°C and dissected later (0-98 days after collection, mean = 19 days). We recorded the pH of the albumen and used the quotient of embryo mass over total egg mass as a proxy for egg age. Seventy-six eggs contained no embryonic material, and of the remainder, only 13 were estimated to be more than four days old based on embryo mass (maximum estimated age = seven days for one egg). Excluding eggs that showed any signs of embryonic development from our analyses did not change our main conclusions. Therefore all results are based on the entire dataset of eggs. The incubation period in all lark species is approximately 12 days [66], with incubation commencing upon laying of the penultimate or last egg (Additional file 1). All eggs were collected during the period March-July 2007-2009.

**Table 3:** Sample sizes, geographic origin and climatic variables for the eggs of the nine larks species used in this study

**Species**	***n***	**nest *n***	**Latitude**	**Longitude**	**Altitude (m)**	**Country**	**P (mm)**	**T (°C)**	**A**_**M**_
Hoopoe lark *Alaemon alaudipes*	18 (8)	9	22° 14’ N	41° 50’ E	1001	Saudi Arabia	42.23	26.22	1.17
Black-crowned finchlark *Eremopterix nigriceps*	3 (1)	3	21° 15’ N	40° 41’ E	1489	Saudi Arabia	64.16	22.40	1.98
Crested lark *Galerida cristata*	1 (0)	1	21° 15’ N	40° 41’ E	1489	Saudi Arabia	64.16	22.40	1.98
Red-capped lark *Calandrella cinerea*	12 (5)	7	0° 52’ S	36° 23’ E	2038	Kenya	193.96	20.72	6.31
	2 (0)	1	0° 37’ S	36° 28’ E	2456	Kenya	275.83	16.07	10.58
Horned lark *Eremophila alpestris*	4 (2)	4	37° 10’ N	72° 53’ E	4084	Afghanistan	78.28	5.88	4.93
	7 (2)	7	37° 24’ N	73° 30’ E	4122	Afghanistan	142.19	1.40	12.48
	1 (0)	1	40° 02’ N	83° 09’ W	284	USA	206.05	13.24	8.86
Hume’s short-toed lark *Calandrella acutirostris*	3 (0)	3	37° 11’ N	72° 49’ E	4401	Afghanistan	78.28	5.88	4.93
	9 (6)	7	37° 25’ N	76° 39’ E	4129	Afghanistan	37.20	5.53	2.40
	1 (0)	1	37° 18’ N	73° 03’ E	3707	Afghanistan	119.09	-2.28	15.42
Oriental skylark *Alauda gulgula*	1 (0)	1	37° 01’ N	72° 41’ E	2802	Afghanistan	86.09	4.15	6.09
Skylark *Alauda arvensis*	29 (13)	14	52° 55’ N	6° 15’ E	10	Netherlands	302.92	11.27	14.24
Woodlark *Lullula arborea*	33 (13)	21	52° 55’ N	6° 15’ E	10	Netherlands	219.47	9.90	11.03

*n*: number of eggs (in brackets, number showing some degree of embryonic development); nest *n:* number of nests. The climatic variables are mean breeding season values for precipitation (P), temperature (T) and the aridity index A_M_ (P/T + 10). A lower value of A_M_ indicates a more arid environment. Altitude values are taken from Google Earth [67].

We measured lysozyme activities by recording the rate of change in optical density (OD, 450 nm) following addition of 200 μl of a 1.0 mg ml^-1^ solution of *Micrococcus lysodeikticus* (M3770) in potassium phosphate buffer (pH 7.0, 100 mM) to microplate wells containing 50 μl of albumen. We used a spectrophotometric microplate reader (VersaMax, Molecular Devices, Sunnyvale, CA, USA) to record OD every ten seconds for 60 minutes at 25°C, with each sample run at two dilutions (see Additional file 2 for more information). Standards of 50 μl purified chicken egg white lysozyme (L6876; over the range 0.04-0.004 mg ml^-1^) were also run in duplicate. We recorded the time (T75, in seconds) at which OD had decreased to 75% of the OD of a negative control (potassium phosphate buffer only). We then used a standard curve relating T75 to lysozyme concentration of the standards to calculate mean lysozyme activities (mg ml^-1^) of the two sample dilutions. We measured ovotransferrin activities (mg ml^-1^) as described in [68], using 10 μl of albumen instead of plasma. All chemicals were purchased from Sigma-Aldrich (St Louis, MO, USA).

### Climatic data and indices of environmental aridity

We obtained high-resolution (0.5 × 0.5 degree – approximately 55 × 55 km) gridded data on climatic variables for the period 1901-2009 from the Climatic Research Unit time series dataset (CRU TS 3.1) [69]. Seasonal variation in environmental and bird-derived microbial abundances [70] suggests that climatic conditions immediately preceding and during egg laying and incubation might have the most influence on the potential for trans-shell infection. Therefore, for each species and location we calculated mean values for precipitation (P, mm) and temperature (T, °C) for the period covering the month prior to that in which the first egg was laid, up to and including the month in which the last egg was laid. We used these climatic variables to calculate an index of aridity, de Martonne’s aridity index A_M_ (P/T + 10) [71]. Low values of A_M_ indicate arid conditions, whereas higher values are associated with increasingly mesic environments.

### Analyses

We performed regression analyses to investigate relationships between antimicrobial protein concentrations of eggs and climatic variables. First, we tested whether we needed to take into account potential non-independence among species due to common ancestry. To do this we calculated mean values (based on mean values per nest) for each population (i.e. per species per location; Table 4). We then used a phylogenetic generalised least squares (pgls) approach [72] to simultaneously estimate maximum likelihood values of the parameter λ and test for phylogenetic signal in the model residuals (Pagel’s lambda) [73]. A value of λ = 0 indicates no phylogenetic signal, whereas λ = 1 suggests that trait evolution is consistent with a strong effect of phylogeny. We based our phylogeny on the recently prepared phylogenetic tree of larks [74]. For the three species with multiple sampled populations we added branches to the tree for each population to create polytomies, with the branch length for each population within a species set to zero (Additional file 3). There was no evidence of phylogenetic signal in any of our datasets (λ never differed significantly from zero) and so we proceeded by constructing a candidate set of general linear mixed models using all the data in our dataset, rather than mean values per nest and per population. First, all predictor variables were converted to standardized (z) scores, to take account of the fact that different variables were measured on different scales [50]. Models included single and combinations of the fixed effects mean precipitation, mean ambient temperature, the interaction of precipitation and temperature, A_M_, and egg albumen pH, and with nest of origin nested within population as random effects. We determined the relative strength of support for each model by calculating Akaike’s Information Criterion corrected for small sample sizes (AIC_c_) and AIC_c_ model weights [75]. Model averaging was used to derive parameter estimates and standard errors. We also calculated the relative importance of each explanatory variable and associated 95% confidence intervals by summing the Akaike weights over all models in which the variable appeared [75]. All analyses were conducted using R, version 2.15.2 [76], with packages caper (pgls [77]), lme4 (model generation [78]), arm (variable standardisation [79]), and MuMIn (model selection and model averaging [80]).

**Table 4:** Mean antimicrobial protein concentrations and albumen pH for the eggs of nine lark species

**Species**	**Lysozyme (mg ml**^**-1**^**)**	**Ovotransferrin (mg ml**^**-1**^**)**	**Albumen pH**
Hoopoe lark *Alaemon alaudipes*	3.03 (0.85)	14.67 (3.55)	7.6 (0.3)
Black-crowned finchlark *Eremopterix nigriceps*	2.72 (1.47)	20.38 (6.21)	7.8 (0.5)
Crested lark *Galerida cristata*	3.66	5.46	8.5
Red-capped lark *Calandrella cinerea*	3.39 (0.60)	12.57 (2.26)	8.2 (0.3)
	1.73	10.27	6.7
Horned lark *Eremophila alpestris*	1.18 (0.26)	17.30 (2.98)	6.8 (0.1)
	1.08 (0.39)	21.32 (5.60)	7.0 (0.3)
	0.98	12.02	7.9
Hume’s short-toed lark *Calandrella acutirostris*	1.24 (0.39)	15.69 (0.97)	6.8 (0.2)
	0.88 (0.32)	25.45 (3.09)	6.9 (0.1)
	1.43	15.80	7.9
Oriental skylark *Alauda gulgula*	2.09	8.34	9.4
Skylark *Alauda arvensis*	1.22 (0.35)	14.61 (1.76)	7.8 (0.1)
Woodlark *Lullula arborea*	1.63 (0.28)	15.83 (2.04)	7.9 (0.2)

The values are calculated from mean values per nest for each population, with standard errors (where appropriate) shown in brackets.

## Competing interests

The authors declare that they have no competing interests.

## Authors’ contributions

NPCH and BIT conceived the study. NPCH and KH conducted the protein concentration assays with the assistance of KDM. NPCH performed the statistical analyses. NPCH wrote the manuscript with the help of BIT and KDM. All authors assisted with obtaining samples. All authors read and approved the final manuscript.

## Supplementary Material

Additional file 1 Information on clutch size and onset of incubation in the lark species in this study. Click here for file

Additional file 2 Additional methods and analysis details for measurement of lysozyme activity in albumen. Click here for file

Additional file 3 Phylogenetic tree illustrating the relationships between the species examined in this study. Click here for file
