# Supplementary material for: Are antimicrobial defences in bird eggs related to climatic conditions associated with risk of trans-shell microbial infection?
Source: Front Zool. 2014 Jul 2;11:49. doi: 10.1186/1742-9994-11-49 (PMC4107615; doi:10.1186/1742-9994-11-49)
Supplement: Additional file 1 — Information on clutch size and onset of incubation in the lark species in this study. [file 1742-9994-11-49-S1.pdf]

Are antimicrobial defences in bird eggs related to climatic conditions associated with risk of trans-shell microbial infection?

Nicholas P.C. Horrocks*, Kathryn Hine, Arne Hegemann, Henry K. Ndithia, Mohammed Shobrak, Stéphane Ostrowski, Joseph B. Williams, Kevin D. Matson & B. Irene Tieleman

*Author for correspondence (nh415@cam.ac.uk)

Additional file 1:

Information on clutch size and onset of incubation in the lark species in this study.

Species	Mean clutch size (range)	Incubation onset*	Data source
Hoopoe lark *Alaemon alaudipes*	2.99 (2-4)	last or penultimate	Own data, [1,3]
Black-crowned finchlark *Eremopterix nigriceps*	2.57 (2-3)	penultimate	[1-3]
Crested lark *Galerida cristata*	4.15 (3-5)	last or penultimate	Own data, [1]
Red-capped lark *Calandrella cinerea*	2.02 (2-3)	last	Own data
	2.00 (2)	last	Own data
Horned lark *Eremophila alpestris*	2.13 (2-5)	last	[2,6]
	2.13 (2-5)	last	[2,6]
	3.14 (2-5)	last	[2,3,6]
Hume’s short-toed lark *Calandrella acutirostris*	2.92 (2-4)	last	[2,3]
	2.92 (2-4)	last	[2,3]
	2.92 (2-4)	last	[2,3]
Oriental skylark *Alauda gulgula*	3.00 (2-5)	last	[2,3]
Skylark *Alauda arvensis*	3.56 (2-5)	last	[4,5]
Woodlark *Lullula arborea*	4.02 (2-6)	last	Own data

***** Incubation onset refers to whether incubation of the clutch commences upon laying of the last or penultimate egg in the clutch.

References

1. Tieleman BI, Williams JB, Visser GH: (2004) **Energy and water budgets of larks in a life history perspective: parental effort varies with aridity.** *Ecology* 2004, **85**:1399-1410.
2. Cramp S: **Tyrant Flycatchers to Thrushes.** In: *Handbook of the Birds of Europe, the Middle East and North Africa: The Birds of the Western Palearctic.* Edited by Cramp S. Oxford: Oxford University Press; 1998.
3. del Hoyo J, Elliott A, Christie DA: **Cotingas to Pipits and Wagtails.** In *Handbook of the Birds of the World, vol. 9.* Barcelona: Lynx Edicions; 2004.
4. Hegemann A, Matson KD, Both C, Tieleman BI: **Immune function in a free-living bird varies over the annual cycle, but seasonal patterns differ between years.** *Oecologia* 2012, **170**:605-618.
5. Hegemann A, Matson KD, Flinks H, Tieleman BI: **Offspring pay sooner, parents pay later: Experimental manipulation of body mass reveals trade-offs between immune function, reproduction and survival.** *Front Zool* 2013, **10**:77.
6. Beason RC, Franks EC: **Breeding behavior of the Horned Lark.** *Auk* 1974, **91**:65-74.
