# Supplementary material for: Are antimicrobial defences in bird eggs related to climatic conditions associated with risk of trans-shell microbial infection?
Source: Front Zool. 2014 Jul 2;11:49. doi: 10.1186/1742-9994-11-49 (PMC4107615; doi:10.1186/1742-9994-11-49)
Supplement: Additional file 2 — Additional methods and analysis details for measurement of lysozyme activity in albumen. [file 1742-9994-11-49-S2.pdf]

Are antimicrobial defences in bird eggs related to climatic conditions associated with risk of trans-shell microbial infection?

Nicholas P.C. Horrocks*, Kathryn Hine, Arne Hegemann, Henry K. Ndithia, Mohammed Shobrak, Stéphane Ostrowski, Joseph B. Williams, Kevin D. Matson & B. Irene Tieleman

*Author for correspondence (nh415@cam.ac.uk)

Additional file 2:

Additional methods and analysis details for measurement of lysozyme activity in albumen.

Lysozyme microplate absorbance assay

The relationship between lysozyme concentration and optical density change (enzymatic activity against *Micrococcus lysodeikticus* in our assay) is not linear. Higher concentrations of lysozyme can form an insoluble complex with the substrate [1,2], resulting in a slowing of the reaction rate, and an increase, rather than a decrease, in optical density over time. Therefore, we ran samples at two dilutions (typically 1:20 and 1:40, diluted with 100 mM potassium phosphate buffer, pH 7.0) in duplicate and checked that the more dilute sample caused the reaction mixture to clear more slowly than the more concentrated sample. If this was not the case then the sample was further diluted and re-run. If a sample did not reach T75 within 60 minutes, then a less-diluted sample was re-run and/or the ratio of sample to *M. lysodeikticus* substrate was adjusted to increase the amount of sample used, while keeping the concentration of *M. lysodeikticus* constant.

Studies measuring lysozyme concentration in albumen or plasma using *M. lysodeikticus* as a substrate have typically followed the format of Osserman and Lawlor [3] by including NaCl in the assay mix and running the assay under slightly acidic conditions (e.g. [4-6]). These conditions catalyse the enzymatic activity of chicken egg lysozyme, but lysozymes from eggs of other bird species may have different optimal reaction conditions [7]. To avoid species-specific assay conditions and thereby obtain measurements of lysozyme concentration (or more specifically, lysozyme activity) that better reflect the biologically relevant conditions 'in ovo', we ran the assay at neutral pH and without the addition of NaCl. A pool of chicken egg albumen from three chicken eggs was run in duplicate as a standard on all plates to assess intra- and inter-assay variation (Table 1).

References

1. Friedberg I, Avigad G: **High lysozyme concentration and lysis of *Micrococcus lysodeikticus*.** *BBA-Gen Subjects* 1966, **127**:532-535.
2. Wilcox FH, Daniel LJ: **Reduced lysis at high concentrations of lysozyme.** *Arch Biochem Biophys* 1954, **52**:305-312.
3. Osserman EF, Lawlor DP: **Serum and urinary lysozyme (muramidase) in monocytic and monomyelocytic leukemia.** *J Exp Med* 1966, **124**:921-52.
4. D'Alba L, Shawkey MD, Korsten P, Vedder O, Kingma SA, Komdeur J, Beissinger SR: **Differential deposition of antimicrobial proteins in Blue Tit (Cyanistes caeruleus) clutches by laying order and male attractiveness.** *Behav Ecol Sociobiol* 2010, **64**: 1037-1045.
5. Saino N, Dall'ara P, Martinelli R, Møller AP: **Early maternal effects and antibacterial immune factors in the eggs, nestlings and adults of the barn swallow.** *J Evol Biol* 2002, **15**:735-743.
6. Shawkey MD, Kosciuch KL, Liu M, Rohwer FC, Loos ER, Wang JM, Beissinger SR: **Do birds differentially distribute antimicrobial proteins within clutches of eggs?** *Behav Ecol* 2008, **19**:920-927.
7. Wellman-Labadie O, Picman J, Hincke MT: **Avian antimicrobial proteins: structure, distribution and activity.** *World Poultry Sci J* 2007, **63**:421-438.

Table 1. Within- and among-plate assay variation for the measurement of lysozyme in egg albumen.

Data are based on a pool of three chicken eggs, run in duplicate on 36 assay microplates. Plates were run over a period of three months. sd: standard deviation; se: standard error; cv: coefficient of variation.

			sd			se			cv		
**Variation**	***n***	**mean**	**mean**	**min**	**max**	**mean**	**min**	**max**	**mean**	**min**	**max**
Within-assay	36 repeats	6.45	0.68	0.00	2.34	0.48	0.00	1.65	0.13	0.00	0.30
Among-assay	36 repeats	6.45	1.44			0.24			0.22		
