# Supplementary material for: Are antimicrobial defences in bird eggs related to climatic conditions associated with risk of trans-shell microbial infection?
Source: Front Zool. 2014 Jul 2;11:49. doi: 10.1186/1742-9994-11-49 (PMC4107615; doi:10.1186/1742-9994-11-49)

Are antimicrobial defences in bird eggs related to climatic conditions associated with risk of trans-shell microbial infection?

Nicholas P.C. Horrocks*, Kathryn Hine, Arne Hegemann, Henry K. Ndithia, Mohammed Shobrak, Stéphane Ostrowski, Joseph B. Williams, Kevin D. Matson & B. Irene Tieleman

*Author for correspondence: nh415@cam.ac.uk

Additional file 3:

A phylogenetic tree illustrating the relationships between the species examined in this study.

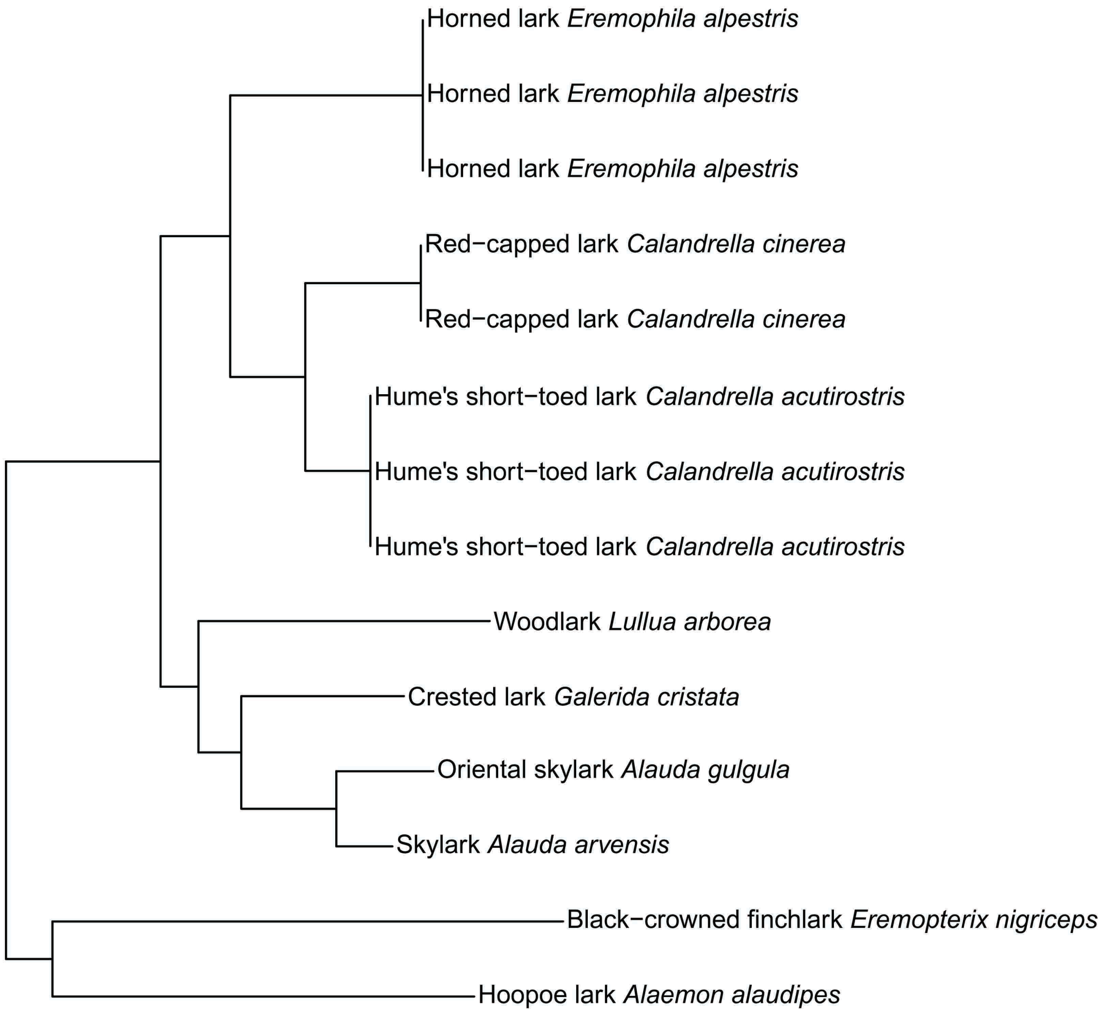

Supplement: Additional file 3 — Phylogenetic tree illustrating the relationships between the species examined in this study. [file 1742-9994-11-49-S3.pdf]
